# Medication use in pediatric patients with covid-19 hospitalized in a referral hospital in Lima, Peru, 2020 - 2022

**DOI:** 10.17843/rpmesp.2023.401.12326

**Published:** 2023-03-31

**Authors:** Medalit Luna-Vilchez, Jhonatan R. Mejia, Zhamanda N. Ortiz-Benique, Mitsi Santiago-Abal, Alvaro Taype-Rondan

**Affiliations:** 1 Instituto Nacional de Salud del Niño San Borja, Lima, Peru. Instituto Nacional de Salud del Niño San Borja Lima Peru; 2 Universidad Científica del Sur, Lima, Peru. Universidad Científica del Sur Universidad Científica del Sur Lima Peru; 3 EviSalud - Evidencias en Salud, Lima, Peru. EviSalud - Evidencias en Salud Lima Peru; 4 Faculty of medicine, Universidad Nacional de San Agustín de Arequipa, Arequipa, Peru. Universidad Nacional de San Agustín Faculty of medicine Universidad Nacional de San Agustín de Arequipa Arequipa Peru; 5 Research Unit for Health Evidence Generation and Synthesis, Universidad San Ignacio de Loyola, Lima, Peru. Universidad San Ignacio de Loyola Research Unit for Health Evidence Generation and Synthesis Universidad San Ignacio de Loyola Lima Peru

**Keywords:** COVID-19, children, drugs, Peru

## Abstract

This study aimed to describe the use of medication in pediatric patients with COVID-19 hospitalized at the National Children’s Health Institute - San Borja (INSN-SB) in Lima, Peru, between March 2020 and March 2022. Therefore, we conducted a descriptive, observational study to evaluate the use of six medications: corticosteroids, ceftriaxone, azithromycin, immunoglobulin, hydroxychloroquine, and ivermectin. We compared the results among subpopulations defined according to the COVID-19 pandemic waves. We included 421 pediatric patients. Corticosteroids were prescribed to 40.4%, ceftriaxone to 35.6%, azithromycin to 12.1%, immunoglobulin to 3.6%, hydroxychloroquine to 2.4%, and ivermectin to 0.5% of the patients. The use of ceftriaxone and azithromycin was less frequent during the third wave. Hydroxychloroquine was used almost exclusively during the first wave. Only patients with pediatric multisystem inflammatory syndrome used immunoglobulin. Overall, the prescription of the evaluated medications decreased during the third wave.

## INTRODUCTION

Worldwide, the pharmacological management of COVID-19 has been constantly modified based on research ^1^. Although certain drugs were widely used during the first wave of the pandemic, several of them were not used during the following waves ^2^^,^^3^.

The Peruvian Ministry of Health established guidelines for the management of patients with COVID-19. At the beginning of the pandemic, these guidelines included the use of ivermectin, chloroquine, hydroxychloroquine, and antibiotics ^4^, which were removed in later updates based on studies conducted and disseminated worldwide ^5^. As the pandemic progressed, Peruvian clinical practice guidelines and guidelines on the treatment of COVID-19 in children were also published ^6^^,^^7^. Decision-making was more complex in these cases due to the scarce evidence of clinical trials evaluating the efficacy of treatment in children ^8^.

A systematic review on the management of COVID-19 in children, which included articles through June 2021, evaluated 97 studies and reported a high prevalence of use of anti-inflammatory medications (including corticosteroids) and a low use of antibiotics and ivermectin ^8^. However, the authors did not report variation in treatment trends over time. In Peru, a study of 100 pediatric patients with possible COVID-19 diagnosis in a Lima hospital during the first wave of the pandemic reported that eight out of ten patients received antibiotic therapy ^9^. However, the variation of these treatments in subsequent waves has not been explored in Peru.

Studying the patterns of drug use for COVID-19 in the pediatric population will allow us to understand how the decision-making process for this disease evolved. This information will be useful to estimate what could happen in the face of future diseases; as well as to establish guidelines to guide evidence-based decision making for the benefit of patients.

Therefore, our study aimed to describe the use of medication in pediatric patients with COVID-19 hospitalized at the Instituto Nacional de Salud del Niño - San Borja (INSN-SB) in Peru, during the first three waves of the pandemic (March 2020 to March 2022).

**KEY MESSAGES**

Motivation for the study. Therapeutic guidelines for COVID-19 in children changed constantly during the pandemic. In Peru, the variation of the treatment during the different waves of the pandemic has not been studied.

Main findings. During the third wave, there was a greater number of patients with COVID-19; however, these patients had less severe symptoms. The use of ceftriaxone and azithromycin was less frequent during the third wave. The use of immunoglobulin was only found in patients with pediatric inflammatory multisystemic syndrome.

Implications. Determining the patterns of medication use during the COVID-19 pandemic in the pediatric population will allow us to evaluate how the therapeutic decision-making process evolved in this population.

## THE STUDY

We conducted a descriptive observational secondary data study at INSN-SB (Lima, Peru). We included all pediatric patients (< 18 years) hospitalized with a diagnosis of COVID-19 from the onset of the pandemic in Peru (March 2020 to March 2022). COVID-19 was diagnosed by reverse transcription polymerase chain reaction (RT-PCR) or antigen testing. We did not include patients diagnosed with COVID-19 by rapid test (antibody detection), as this test does not determine current COVID-19 infection.

INSN-SB is a highly complex pediatric center in Peru, providing care to patients referred from all over the country. This institution contributed to the management of patients from the beginning of the COVID-19 pandemic, developing local protocols and participating in the development of national protocols for the management of this disease ^10^.

We collected data regarding sociodemographic information, diagnosis and COVID-19 treatment from the Integrated Hospital Management System (SISGalenPlus), a software developed for adequate management of healthcare and administrative information. With this information, each case was assigned to a pandemic wave according to the date of sampling (first wave: April 2020 to November 2020, second wave: December 2020 to November 2021, third wave: December 2021 to March 2022, according to visual inspection of Peruvian statistics available at: https://www.worldometers.info/coronavirus/country/peru/).

Subsequently, we used the epidemiological records of patients diagnosed with COVID-19 to collect the following variables: sex, age, place of diagnosis, date of hospitalization, cause of hospitalization (COVID-19 or other condition), intensive care admission, discharge condition (death or survival), severity status during the course of pediatric COVID-19 disease (asymptomatic, mild, moderate, severe, or pediatric inflammatory multisystem syndrome [PIMS]; using criteria established by WHO) ^11^, and medications prescribed during hospitalization (corticosteroids, ceftriaxone, azithromycin, immunoglobulin, hydroxychloroquine, and ivermectin).

We exported the variables of interest to a Microsoft Excel document. After quality control, the database was imported to the statistical package Stata version 16 (StataCorp., College Station, TX, USA) for analysis. Categorical variables were described with absolute frequencies and percentages. Quantitative variables were described with mean and standard deviation or median and interquartile range (IQR), as appropriate. We compared results between subpopulations according to the COVID-19 pandemic wave, using the chi-square test, Fisher’s exact test, or Mann-Whitney U, and considering a p<0.05 as statistically significant.

The study protocol was approved by the INSN-SB ethics committee (certificate 031-2022, project code: PI671). During the study, the anonymity of the patients was respected by using anonymization codes.

## FINDINGS

**Figure 1:**
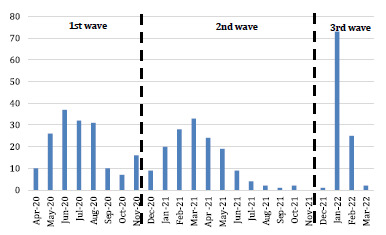
Number of pediatric patients hospitalized with COVID-19 at INSN-SB by month.

We included 421 pediatric patients diagnosed with COVID-19 by RT-PCR and antigenic testing, who were hospitalized at INSN-SB. Figure 1 shows the patient distribution during the first three waves of the pandemic (March 2020 to March 2022). The month with the highest number of hospitalizations was January 2022, during the third wave of COVID-19 in Peru.

The median age was 7 years, 57.7% were male, 27.3% were admitted to the intensive care unit, 5.2% had PIMS and 9.3% died. The proportion of patients hospitalized for COVID-19 as well as the proportion of asymptomatic patients were lower during the third wave (p-value<0.001). The proportion of deaths was higher in the first wave (p-value=0.002) (Table 1).

**Table 1:** Characteristics of pediatric patients hospitalized with COVID-19 at the Instituto Nacional de Salud del Niño - San Borja, overall and for each wave.

Characteristic	Total N=421 n (%)	First wave (April 2020 to November 2020) N=169 n (%)	Second wave (December 2020 to November 2021) N=151 n (%)	Third wave (December 2021 to March 2022) N=101 n (%)	p-value
Male sex	243 (57.7)	96 (56.8)	85 (56.3)	62 (61.4)	0.691^a^
Age in years, Median (IQR)	7 (3 - 11)	6 (3-11)	7 (3-11)	7 (5-11)	0.249 ^b^
COVID-19 diagnosis was made during or after hospitalization at the institute.	356 (84.6)	132 (78.1)	142 (94.0)	82 (81.2)	<0.001^a^
COVID-19 was the cause of hospitalization.	42 (10.0)	7 (4.1)	31 (20.5)	4 (4.0)	<0.001^a^
Intensive care unit	115 (27.3)	51 (30.2)	46 (30.5)	18 (17.8)	0.049 ^a^
Severity of COVID-19					<0.001^a^
Asymptomatic	196 (46.6)	55 (32.5)	71 (47.0)	70 (69.3)	
Mild or moderate	187 (44.4)	100 (59.2)	62 (41.1)	25 (24.8)	
Severe	16 (3.8)	0 (0.0)	10 (6.6)	6 (5.9)	
PIMS	22 (5.2)	14 (8.3)	8 (5.3)	0 (0.0)	
Deceased	39 (9.3)	26 (15.4)	7 (4.6)	6 (5.9)	0.002 ^a^

a Chi-square test, ^b^ Mann-Whitney U test.

IQR: interquartile range; PIMS: pediatric inflammatory multisystem syndrome.

Regarding drug use, 40.4% of patients received corticosteroids, 35.6% received ceftriaxone, 12.1% received azithromycin, 3.6% received immunoglobulin, 2.4% received hydroxychloroquine and 0.5% received ivermectin. According to the severity, two out of ten asymptomatic cases received corticosteroids (28.1%). Likewise, severe cases received mostly corticosteroids (87.5%), ceftriaxone (43.8%), and azithromycin (43.8%); while PIMS cases received mostly immunoglobulin (68.2%) and ceftriaxone (59.1%) (Table 2). The prescription of ceftriaxone and azithromycin was less frequent during the third wave (p<0.001), and more frequent during the second wave (p=0.037). Out of every 10 patients who received hydroxychloroquine, 9 were from the first wave (p=0.002) (Table 3).

**Table 2:** Medications prescribed to hospitalized pediatric patients with COVID-19 at the Instituto Nacional de Salud del Niño - San Borja, by severity and presence of pediatric inflammatory multisystemic syndrome.

Medication	Total (N=421) n (%)	Asymptomatic (N=196) n (%)	Mild or moderate (N=187) n (%)	Severe (N=16) n (%)	PIMS (N=22) n (%)	p-value
Corticoids	170 (40.4)	55 (28.1)	89 (47.6)	14 (87.5)	12 (54.5)	< 0.001 ^a^
Ceftriaxone	150 (35.6)	39 (19.9)	91 (48.7)	7 (43.8)	13 (59.1)	< 0.001 ^a^
Azithromycin	51 (12.1)	8 (4.1)	34 (18.2)	7 (43.8)	2 (9.1)	< 0.001 ^a^
Immunoglobulin	15 (3.6)	0 (0.0)	0 (0.0)	0 (0.0)	15 (68.2)	< 0.001 ^b^
Hydroxychloroquine	10 (2.4)	4 (2.0)	5 (2.7)	0 (0.0)	1 (4.5)	0.689 ^b^
Ivermectin	2 (0.5)	1 (0.5)	0 (0.0)	1 (6.3)	0 (0.0)	0.077 ^b^

a Chi-square test, ^b^ Fisher’s exact test.

**Table 3:** Medications provided to hospitalized pediatric patients with COVID-19 at the Instituto Nacional de Salud del Niño - San Borja by wave.

Medications	First wave (April 2020 to November 2020) (N=169) n (%)	Second wave (December 2020 to November 2021) (N=151) n (%)	Third wave (December 2021 to March 2022) (N=101) n (%)	p-value
Corticoids	62 (36.7)	72 (47.7)	36 (35.6)	0.073 ^a^
Ceftriaxone	63 (37.3)	70 (46.4)	17 (16.8)	<0.001 ^a^
Azithromycin	18 (10.7)	26 (17.2)	7 (6.9)	0.037 ^a^
Immunoglobulin	11 (6.5)	8 (5.3)	1 (1.0)	0.087 ^b^
Hydroxychloroquine	9 (5.3)	0 (0.0)	1 (1.0)	0.002 ^b^
Ivermectin	1 (0.6)	1 (0.7)	0 (0.0)	0.999 ^b^

a Chi-square test, ^b^ Fisher’s exact test.

## DISCUSSION

Our study evaluated the use of six drugs, of which the most commonly used were corticosteroids (40.4%), ceftriaxone (35.6%) and azithromycin (12.1%). Previous articles evaluated different drugs and reported different results. A study in an Indian hospital, conducted from June 2020 to June 2021, found that antibiotics (77%) were more prescribed than dexamethasone (9%), remdesivir (8%) and immunoglobulin (6%) ^12^. Another study, conducted in 25 European countries during April 2020, reported higher prescription of hydroxychloroquine (7%), corticosteroids (4%) and remdesivir (3%) ^13^. On the other hand, a Peruvian study, conducted between March and August 2020, found that 88.2% of hospitalized children received antibiotics, 29.4% corticosteroids and 29.4% ivermectin ^9^.

We found that the use of the most commonly prescribed drugs (corticosteroids, ceftriaxone and azithromycin) decreased in the third wave (although this was not statistically significant for corticosteroids). Previous studies have reported heterogeneous results in children. A Turkish study reported that the use of azithromycin and hydroxychloroquine decreased when comparing the second wave with the first wave, whereas the use of antivirals, immunoglobulin, and corticosteroids increased ^14^. In contrast, a study from India found no difference when comparing the drugs used during the first and second waves ^12^. This heterogeneity may be due to the fact that these studies only compared the first two waves (up to June 2021), and it is possible that by that date the management of COVID-19 was not yet been standardized in some places.

Although corticosteroids have been considered beneficial in cases of moderate or severe COVID-19, corticosteroids should not be used in children with asymptomatic COVID-19 infection ^7^. Our results showed that 40.4% of pediatric patients with COVID-19 received in-hospital corticosteroids, including 28.1% of asymptomatic cases. However, a systematic review in children found that most asymptomatic cases did not receive any treatment ^8^. Our findings suggest the need for further research, since it is possible that some of our participants may have required corticosteroids due to concomitant conditions.

Antibiotics were frequently used during the beginning of the pandemic because bacterial superinfection was thought to be common, and that antibiotics would modulate the progression of the disease. However, massive antibiotic therapy was later proscribed because of reports of low bacterial coinfection ^15^. In our study, ceftriaxone and azithromycin were used in 35.6% and 12.1% of patients, although these figures decreased over time. Similarly, a report from Spain found that 54.2% of hospitalized children received antibiotic therapy; they also found a negative trend of prescription for ceftriaxone and azithromycin from the end of 2020 ^16^. This could be due to the fact that we only assessed the use of two antibiotics, while other studies evaluated a greater number of this type of drugs.

The prescription of immunoglobulins was found to be within the norm for patients with PIMS, due to indirect evidence of possible benefit in other conditions, such as Kawasaki disease, mast cell activation syndrome, among others ^16^. In our study, 68.2% of children with PIMS received immunoglobulins. This was lower during the third wave, where no cases of PIMS were reported. A study from Turkey reported no prescriptions of immunoglobulins during the first wave, however, 4.3% of children were treated with this medication during the second wave with half of them being patients with PIMS ^14^.

Early during the pandemic, hydroxychloroquine and ivermectin were recommended for the management of COVID-19 ^4^, leading to their inappropriate prescription in several countries. In Peru, a report showed that 23.5% of hospitalized children received hydroxychloroquine and 29.4%, ivermectin ^9^. However, in the present study only 2.4% and 0.5% of hospitalized children received hydroxychloroquine and ivermectin, respectively, and their prescription during the second and third waves was almost nonexistent.

The use of hydroxychloroquine decreased as the waves went by, this may be related to the evidence against its use, which showed more adverse events and no benefit ^18^. In addition, the early issuance of the institutional technical guide for treatment of pediatric COVID-19 by INSN-SB did not recommend any of these drugs due to the limited evidence in favor of their use ^10^. Despite this, the Peruvian Ministry of Health continued to recommend this drug until late in the pandemic, when it was decided to discontinue its inclusion in national protocols ^5^.

One of the limitations of this study is that it used secondary data, therefore, it is possible that some of the prescribed data were not administered. However, we consider that the prescription was the most relevant action for our research question, as it reflects the intention to use the medications evaluated. Likewise, it is possible that certain medications may have been prescribed as part of the treatment for other comorbidities and not as part of the treatment for COVID-19, a limitation shared with similar studies ^2^^,^^8^.

To our knowledge, this is the first study in Peru to collect data on children hospitalized throughout three waves of the COVID-19 pandemic, allowing comparisons by date and severity.

A greater number of pediatric patients were hospitalized at INSN-SB with a diagnosis of COVID-19 during the third wave of the pandemic, but these were less severe cases. More than one third received corticosteroids and ceftriaxone, one in ten received azithromycin, and less than 4% received immunoglobulin, hydroxychloroquine or ivermectin. The prescription of the evaluated medications decreased during the third wave, when compared to the first and second waves.
