# Supplementary material for: Medication use in pediatric patients with covid-19 hospitalized in a referral hospital in Lima, Peru, 2020 - 2022
Source: Rev Peru Med Exp Salud Publica. 2023 Mar 31;40(1):73–8. doi: 10.17843/rpmesp.2023.401.12326 (PMC10959514; doi:10.17843/rpmesp.2023.401.12326)
Supplement: Translation

# Translation: Uso de medicamentos en pacientes pediátricos con COVID-19 hospitalizados en un hospital de referencia de Lima, Perú, 2020-2022

**Authors:** Medalit Luna-Vilchez, Jhonatan R. Mejia, Zhamanda N. Ortiz-Benique, Mitsi Santiago-Abal, Alvaro Taype-Rondan

**Affiliations:** 1 Instituto Nacional de Salud del Niño San Borja, Lima, Perú.; 2 Universidad Científica del Sur, Lima, Perú.; 3 EviSalud - Evidencias en Salud, Lima, Perú.; 4 Facultad de Medicina, Universidad Nacional de San Agustín de Arequipa, Arequipa, Perú.; 5 Unidad de Investigación para la Generación y Síntesis de Evidencias en Salud, Universidad San Ignacio de Loyola, Lima, Perú.

**Keywords:** COVID-19, niños, medicamentos, Perú

## Abstract

El objetivo del estudio fue describir el uso de medicamentos en pacientes pediátricos con COVID-19 hospitalizados en el Instituto Nacional de Salud del Niño - San Borja (INSN-SB) de Lima, Perú, entre marzo del 2020 y marzo del 2022. Por ello, realizamos un estudio observacional descriptivo y evaluamos el uso de seis medicamentos: corticoides, ceftriaxona, azitromicina, inmunoglobulina, hidroxicloroquina e ivermectina. Finalmente, comparamos los resultados entre subpoblaciones definidas según la ola de la pandemia por COVID-19. Incluimos 421 pacientes pediátricos. Al 40,4% se le indicó corticoides, 35,6% ceftriaxona, 12,1% azitromicina, 3,6% inmunoglobulina, 2,4% hidroxicloroquina y 0,5% ivermectina. El uso de ceftriaxona y azitromicina fue menos frecuente durante la tercera ola. El uso de hidroxicloroquina fue casi exclusivo de la primera ola. El uso de inmunoglobulina solo se observó en pacientes con síndrome inflamatorio multisistémico pediátrico. La indicación de los medicamentos evaluados fue en general menor en la tercera ola.

## INTRODUCCIÓN

A nivel mundial, el manejo farmacológico de COVID-19 fue modificándose en base a los resultados de las investigaciones que se fueron realizando al respecto ^1^. Si bien durante la primera ola de la pandemia se solían usar masivamente ciertos medicamentos, varios de estos cayeron en desuso durante las siguientes olas ^2^^,^^3^. 

El Ministerio de Salud del Perú estableció lineamientos sobre el manejo de pacientes con COVID-19. Al inicio de la pandemia, estos lineamientos incluyeron el uso de ivermectina, cloroquina, hidroxicloroquina, y antibióticos ^4^, que fueron retirados en posteriores actualizaciones en base a los estudios realizados y difundidos a nivel mundial ^5^. Al avanzar la pandemia, también se publicaron normas y guías de práctica clínica peruanas sobre el tratamiento de COVID-19 en niños ^6^^,^^7^. En estos casos, las decisiones fueron más complejas debido a la escasa evidencia de ensayos clínicos que evalúen la eficacia de tratamientos en niños ^8^. 

Una revisión sistemática sobre el manejo de COVID-19 en niños, que incluyó artículos hasta junio del 2021, evaluó 97 estudios y reportó mayor prevalencia de uso de medicamentos antiinflamatorios (incluidos los corticoides) y menor uso de antibióticos e ivermectina ^8^. Sin embargo, los autores no reportaron la variación de las tendencias de prescripción en el tiempo. En el Perú, un estudio realizado en 100 pacientes pediátricos con diagnóstico posible de COVID-19 en un hospital de Lima durante la primera ola de la pandemia reportó que ocho de cada diez pacientes recibieron antibioticoterapia ^9^. Sin embargo, la variación de estos tratamientos en las olas siguientes no ha sido explorada en el Perú.

Estudiar los patrones de uso de medicamentos para COVID-19 en población pediátrica nos permitirá registrar cómo evolucionó el proceso de toma de decisiones para esta enfermedad. Esta información será útil para estimar lo que podría suceder ante futuras enfermedades que aparezcan; así como establecer lineamientos que permitan guiar la toma de decisiones basada en evidencias en beneficio de los pacientes.

Por ello, el presente estudio tuvo por objetivo describir el uso de medicamentos en pacientes pediátricos con COVID-19 hospitalizados en el Instituto Nacional de Salud del Niño - San Borja (INSN-SB) de Perú, durante las tres primeras olas de la pandemia (marzo del 2020 a marzo del 2022).

**MENSAJES CLAVE**

Motivación para realizar el estudio. Las guías terapéuticas acerca de COVID-19 en población pediátrica variaron constantemente durante la pandemia. En el Perú, la variación de estos tratamientos en las diferentes olas de la pandemia no ha sido estudiada.

Principales hallazgos. Durante la tercera ola hubo un mayor número de pacientes con COVID-19; sin embargo, estos presentaron cuadros menos severos. El uso de ceftriaxona y azitromicina fue menos frecuente durante la tercera ola. El uso de inmunoglobulina solo se observó en pacientes con síndrome inflamatorio multisistémico pediátrico.

Implicancias. Determinar los patrones de uso de medicamentos durante la pandemia por COVID-19 en población pediátrica nos permitirá evaluar cómo evolucionó el proceso de toma de decisiones terapéuticas en esta población.

## EL ESTUDIO

Realizamos un estudio observacional descriptivo de datos secundarios en el INSN-SB (Lima, Perú). Incluimos a todos los pacientes pediátricos (< 18 años) hospitalizados con diagnóstico de COVID-19 desde el inicio de la pandemia en Perú (marzo del 2020 hasta marzo del 2022). El diagnóstico de COVID-19 se realizó mediante la prueba de reacción en cadena de la polimerasa con transcripción inversa (RT-PCR) o prueba antigénica. No incluimos a los pacientes con diagnóstico de COVID-19 por prueba rápida (detección de anticuerpos), pues esta prueba no determina una infección actual por COVID-19.

El INSN-SB es un centro pediátrico de alta complejidad a nivel nacional, que brinda atención a pacientes referidos de todo el país. Esta institución contribuyó con el manejo de pacientes desde el inicio de la pandemia por COVID-19, desarrollando protocolos locales y participando en el desarrollo de protocolos nacionales del manejo de esta enfermedad ^10^.

Obtuvimos los datos sociodemográficos, diagnóstico y tratamiento de COVID-19 a través del Sistema Integrado de Gestión Hospitalaria (SISGalenPlus), *software* desarrollado para un adecuado manejo de información asistencial y administrativa. Con esta información, usando la fecha de toma de muestra, identificamos la ola de la pandemia a la que correspondió cada caso (primera ola: abril 2020 a noviembre 2020, segunda ola: diciembre 2020 a noviembre 2021, tercera ola: diciembre 2021 a marzo 2022, según inspección visual de las estadísticas peruanas disponibles en: https://www.worldometers.info/coronavirus/country/peru/). 

Posteriormente, utilizamos los registros epidemiológicos de los pacientes con diagnóstico de COVID-19 para recolectar las siguientes variables: sexo, edad, lugar de realización de diagnóstico, fecha de hospitalización, causa de hospitalización (COVID-19 u otra condición), ingreso a cuidados intensivos, condición de egreso (muerte o supervivencia), estado de gravedad durante el curso de la enfermedad por COVID-19 en pediatría (asintomático, leve, moderado, severo, o síndrome inflamatorio multisistémico pediátrico [PIMS]; usando los criterios establecidos por la OMS) ^11^, y medicamentos indicados durante la hospitalización (corticoides, ceftriaxona, azitromicina, inmunoglobulina, hidroxicloroquina e ivermectina). 

Exportamos las variables de interés a un documento de Microsoft Excel. Posterior al control de calidad, ingresamos la base de datos al paquete estadístico Stata versión 16 (StataCorp., College Station, TX, EE.UU.) para su análisis. Las variables categóricas fueron descritas con frecuencias absolutas y porcentajes. Las variables cuantitativas fueron descritas con la media y desviación estándar o con la mediana y rango intercuartílico (RIC), según corresponda. Comparamos los resultados entre subpoblaciones definidas según la ola de la pandemia por COVID-19, usando la prueba de chi cuadrado, la prueba exacta de Fisher, o la U de Mann-Whitney, y consideramos un p<0,05 como estadísticamente significativo.

El protocolo del estudio fue aprobado por el comité de ética del INSN-SB (constancia 031-2022, código de proyecto: PI671). Durante el desarrollo del estudio, se respetó el anonimato de los pacientes utilizando códigos para su anonimización.

## HALLAZGOS

Incluimos un total de 421 pacientes pediátricos hospitalizados en el INSN-SB con diagnóstico de COVID-19 por RT-PCR y prueba antigénica. En la figura 1 se observa la distribución de estos pacientes durante las tres primeras olas de la pandemia (marzo del 2020 a marzo del 2022). El mayor número de hospitalizaciones por mes fue registrado en enero del 2022, durante la tercera ola de COVID-19 en el Perú.

**Figura 1:** Número de pacientes pediátricos hospitalizados con COVID-19 en el INSN-SB por mes.

El 57,7% fue de sexo masculino y la mediana de edad fue de siete años. El 27,3% de los casos ingresaron a la unidad de cuidados intensivos, el 5,2% tuvo PIMS y el 9,3% falleció. Durante la tercera ola se encontró una menor proporción de pacientes hospitalizados por COVID-19 así como una menor proporción de pacientes asintomáticos (valor de p<0,001). La proporción de fallecidos fue mayor en la primera ola (valor de p=0,002) (Tabla 1).

**Tabla 1:** Características de los pacientes pediátricos hospitalizados con COVID-19 en el Instituto Nacional de Salud del Niño - San Borja, en total y por cada ola.

Característica	Total N=421 n (%)	Primera ola (abril 2020 a noviembre 2020) N=169 n (%)	Segunda ola (diciembre 2020 a noviembre 2021) N=151 n (%)	Tercera ola (diciembre 2021 a marzo 2022) N=101 n (%)	Valor de p
Sexo masculino	243 (57,7)	96 (56,8)	85 (56,3)	62 (61,4)	0,691^a^
Edad en años, mediana (RIC)	7 (3 - 11)	6 (3-11)	7 (3-11)	7 (5-11)	0,249 ^b^
El diagnóstico de COVID-19 fue realizado durante o después de la hospitalización en el instituto	356 (84,6)	132 (78,1)	142 (94,0)	82 (81,2)	<0,001^a^
COVID-19 fue la causa de la hospitalización	42 (10,0)	7 (4,1)	31 (20,5)	4 (4,0)	<0,001^a^
Unidad de cuidados intensivos	115 (27,3)	51 (30,2)	46 (30,5)	18 (17,8)	0,049 ^a^
Gravedad del cuadro de COVID-19					<0,001^a^
Asintomático	196 (46,6)	55 (32,5)	71 (47,0)	70 (69,3)	
Leve o moderado	187 (44,4)	100 (59,2)	62 (41,1)	25 (24,8)	
Severo	16 (3,8)	0 (0,0)	10 (6,6)	6 (5,9)	
PIMS	22 (5,2)	14 (8,3)	8 (5,3)	0 (0,0)	
Falleció	39 (9,3)	26 (15,4)	7 (4,6)	6 (5,9)	0,002 ^a^

a Prueba de chi cuadrado, ^b^ prueba de U de Mann-Whitney.

RIC: rango intercuartílico, PIMS: síndrome inflamatorio multisistémico pediátrico.

Con respecto al uso de medicamentos, el 40,4% de los pacientes tuvo prescripción de corticoides, el 35,6% recibió ceftriaxona, el 12,1% recibió azitromicina, el 3,6% recibió inmunoglobulina, el 2,4% recibió hidroxicloroquina y el 0,5% recibió ivermectina. Según la gravedad, dos de cada diez casos asintomáticos recibieron corticoides (28,1%). Asimismo, los casos severos recibieron en su mayoría corticoides (87,5%), ceftriaxona (43,8%), y azitromicina (43,8%); mientras que los casos de PIMS recibieron principalmente inmunoglobulina (68,2%) y ceftriaxona (59,1%) (Tabla 2). La indicación de ceftriaxona y azitromicina fue menos frecuente durante la tercera ola (p<0,001), y más frecuente en la segunda ola (p=0,037). De cada 10 pacientes a los que se les indicó hidroxicloroquina, 9 fueron de la primera ola (p=0,002) (Tabla 3).

**Tabla 2:** Medicamentos brindados a los pacientes pediátricos hospitalizados con COVID-19 en el Instituto Nacional de Salud del Niño - San Borja, por nivel de severidad y síndrome inflamatorio multisistémico pediátrico.

Medicamentos	Total (N=421) n (%)	Asintomático (N=196) n (%)	Leve o moderado (N=187) n (%)	Severo (N=16) n (%)	PIMS (N=22) n (%)	Valor de p
Corticoides	170 (40,4)	55 (28,1)	89 (47,6)	14 (87,5)	12 (54,5)	< 0,001 ^a^
Ceftriaxona	150 (35,6)	39 (19,9)	91 (48,7)	7 (43,8)	13 (59,1)	< 0,001 ^a^
Azitromicina	51 (12,1)	8 (4,1)	34 (18,2)	7 (43,8)	2 (9,1)	< 0,001 ^a^
Inmunoglobulina	15 (3,6)	0 (0,0)	0 (0,0)	0 (0,0)	15 (68,2)	< 0,001 ^b^
Hidroxicloroquina	10 (2,4)	4 (2,0)	5 (2,7)	0 (0,0)	1 (4,5)	0,689 ^b^
Ivermectina	2 (0,5)	1 (0,5)	0 (0,0)	1 (6,3)	0 (0,0)	0,077 ^b^

a Prueba de chi cuadrado, ^b^ prueba exacta de Fisher.

**Tabla 3:** Medicamentos brindados a los pacientes pediátricos hospitalizados con COVID-19 en el Instituto Nacional de Salud del Niño - San Borja por ola.

Medicamentos	1ra ola (abril 2020 a noviembre 2020) (N=169) n (%)	2da ola (diciembre 2020 a noviembre 2021) (N=151) n (%)	3ra ola (diciembre 2021 a marzo 2022) (N=101) n (%)	Valor de p
Corticoides	62 (36,7)	72 (47,7)	36 (35,6)	0,073 ^a^
Ceftriaxona	63 (37,3)	70 (46,4)	17 (16,8)	<0,001 ^a^
Azitromicina	18 (10,7)	26 (17,2)	7 (6,9)	0,037 ^a^
Inmunoglobulina	11 (6,5)	8 (5,3)	1 (1,0)	0,087 ^b^
Hidroxicloroquina	9 (5,3)	0 (0,0)	1 (1,0)	0,002 ^b^
Ivermectina	1 (0,6)	1 (0,7)	0 (0,0)	0,999 ^b^

a Prueba de chi cuadrado, ^b^ prueba exacta de Fisher.

## DISCUSIÓN

Nuestro estudio evaluó el uso de seis medicamentos, de los cuales los más usados fueron corticoides (40,4%), ceftriaxona (35,6%) y azitromicina (12,1%). Artículos previos evaluaron distintos medicamentos y reportaron distintos resultados. Un estudio en un hospital hindú, realizado desde junio de 2020 hasta junio de 2021, encontró un mayor uso de antibióticos (77%) que de dexametasona (9%), remdesivir (8%) e inmunoglobulina (6%) ^12^. Otro estudio, realizado en 25 países europeos durante abril 2020, reportó mayor prescripción de hidroxicloroquina (7%), corticoesteroides (4%) y remdesivir (3%) ^13^. Por otro lado, un estudio peruano, realizado entre marzo y agosto de 2020, indicó que 88,2% de los niños hospitalizados recibieron antibióticos, 29,4% corticoides y 29,4% ivermectina ^9^. 

Encontramos que la indicación de los medicamentos más usados (corticoides, ceftriaxona y azitromicina) disminuyó en la tercera ola (aunque esto no fue estadísticamente significativo para los corticoides). Estudios previos han reportado resultados heterogéneos en niños. Un estudio turco reportó que el uso de azitromicina e hidroxicloroquina disminuyó al comparar la segunda ola con la primera, en tanto que el uso de antivirales, inmunoglobulina, y corticoides aumentó ^14^. En contraste, un estudio de la India no encontró diferencias al comparar los medicamentos usados durante la primera y segunda ola ^12^. Esta heterogeneidad puede deberse a que dichos estudios solo compararon las dos primeras olas (hasta junio 2021), siendo posible que para esa fecha en ciertos lugares aún no se haya estandarizado el manejo de la COVID-19.

Si bien los corticoides han sido considerados beneficiosos en casos de COVID-19 moderado o severo, la infección asintomática por COVID-19 no es indicación para el uso de corticoides en niños ^7^. En nuestro estudio, 40,4% de los pacientes pediátricos con COVID-19 recibieron corticoides intrahospitalariamente, incluyendo el 28,1% de los casos asintomáticos. Sin embargo, una revisión sistemática en niños encontró que la mayoría de casos asintomáticos no recibió tratamiento alguno ^8^. Nuestros hallazgos al respecto requieren futuras indagaciones, pues es posible que parte de estos participantes hayan requerido recibir corticoides debido a condiciones concomitantes.

El uso de antibioticoterapia fue frecuente durante el inicio de la pandemia, debido que se sospechaba que la sobreinfección bacteriana podría ser frecuente, y que los antibióticos modularían la progresión de la enfermedad. Sin embargo, la antibioticoterapia masiva fue proscrita debido a los reportes de baja coinfección bacteriana ^15^. En nuestro estudio, la ceftriaxona y la azitromicina fueron usadas en 35,6% y 12,1% de los pacientes, aunque estas cifras fueron disminuyendo con el tiempo. Concordantemente, en España se evidenció que 54,2% de los niños hospitalizados recibieron antibioticoterapia, y una tendencia negativa de prescripción para ceftriaxona y azitromicina a partir de fines del 2020 ^16^. Parte de estas diferencias podrían deberse a que nosotros solo evaluamos el uso de dos antibióticos, mientras otros estudios evaluaron un mayor número de este tipo de medicamentos.

La administración de inmunoglobulinas se encontró dentro de lo habitual para pacientes con PIMS, debido a evidencia indirecta de posible beneficio de otras patologías, tales como enfermedad de Kawasaki, síndrome de activación mastocitaria, entre otras _^17^_. En nuestro estudio, 68,2% de los niños con PIMS recibieron inmunoglobulinas. Esto fue menor en la tercera ola, donde no se reportó ningún caso de PIMS. Un estudio de Turquía no reportó ninguna prescripción durante la primera ola, sin embargo, 4,3% de niños fueron tratados con inmunoglobulinas durante la segunda ola siendo la mitad de ellos pacientes con PIMS ^14^.

La hidroxicloroquina y la ivermectina fueron tempranamente recomendadas para el manejo de COVID-19 ^4^, guiando a su indebida prescripción en diversos países. En Perú, se reportó que 23,5% de los niños hospitalizados recibieron hidroxicloroquina y 29,4% ivermectina ^9^. Sin embargo, en el presente estudio solo 2,4% y 0,5% de los niños hospitalizados recibieron hidroxicloroquina e ivermectina, respectivamente, y su prescripción durante la segunda y tercera ola fue casi inexistente.

El menor uso de hidroxicloroquina a medida que surgían las olas se relacionaría a la evidencia en contra de su uso, que demostraba mayor presencia de eventos adversos y ningun beneficio ^18^. Además, la emisión temprana de la guía técnica institucional de tratamiento de COVID-19 pediátrico del INSN-SB no recomendó ninguno de estos medicamentos debido a la escasa evidencia a favor de su uso ^10^. Pese a ello, el Ministerio de Salud peruano continuó recomendando este medicamento hasta avanzada la pandemia, más tarde se decidió descontinuar su inclusión en los protocolos nacionales ^5^.

El presente estudio tiene algunas limitaciones: al basarse en registros secundarios, es posible que algunos medicamentos indicados no hayan sido administrados. Sin embargo, consideramos que la indicación fue la acción más relevante para nuestra pregunta de investigación, pues refleja la intención de uso de los medicamentos evaluados. Asimismo, es posible que, en algunos pacientes, ciertos medicamentos hayan sido indicados como parte del tratamiento de otras comorbilidades y no como parte del tratamiento para COVID-19, limitación compartida con estudios similares ^2^^,^^8^.

A nuestro conocimiento, éste es el primer estudio en Perú que recopila datos de niños hospitalizados a lo largo de tres olas de la pandemia de COVID-19, permitiendo comparaciones por fechas y por gravedad.

Durante la tercera ola de la pandemia hubo un mayor número de pacientes pediátricos hospitalizados en el INSN-SB con diagnóstico de COVID-19, pero con cuadros clínicos de menor severidad. Más de la tercera parte recibieron corticoides y ceftriaxona, uno de cada diez recibió azitromicina, y menos del 4% recibieron inmunoglobulina, hidroxicloroquina o ivermectina. Encontramos una disminución de la prescripción de los medicamentos evaluados en la tercera ola en comparación con la primera y segunda ola.
